# Thromboembolic events in patients with paroxysmal nocturnal hemoglobinuria (PNH): Real world data of a Greek nationwide multicenter retrospective study

**DOI:** 10.3389/fonc.2023.1128994

**Published:** 2023-03-07

**Authors:** S. Chatzileontiadou, E. Hatjiharissi, M. Angelopoulou, J. V. Asimakopoulos, N. E. Loutsidi, T. Chatzikonstantinou, P. Zikos, A. Bouchla, Z. Bezirgiannidou, E. Kouvata, C. Frouzaki, P. Chaloudis, D. Sotiropoulos, V. Douka, A. Sirigou, E. Mandala, M. Psyllaki, H. A. Papadaki, T. Marinakis, N. A. Viniou, S. Kokkori, F. Kontopidou, A. Skepetari, G. Vassilopoulos, I. Kotsianidis, V. Pappa, C. Lalayanni, I. Baltadakis, S. Delimpassi, M. Pagoni, M. Papaioannou

**Affiliations:** ^1^ Hematology Unit, 1st Department of Internal Medicine, AHEPA University Hospital, Aristotle University of Thessaloniki, Thessaloniki, Greece; ^2^ Department of Hematology, National and Kapodistrian University of Athens, Laikon General Hospital, Athens, Greece; ^3^ Hematology - Lymphomas Department and Bone Marrow Transplant Unit, Evangelismos General Hospital, Athens, Greece; ^4^ Department of Hematology, Bone Marrow Transplantation Unit, G. Papanicolaou Hospital, Thessaloniki, Greece; ^5^ Department of Hematology, General Hospital of Patras “Aghios Andreas”, Patras, Greece; ^6^ Second Department of Internal Medicine and Research Unit, Hematology Unit, University General Hospital, “Attikon”, Athens, Greece; ^7^ Department of Hematology, University Hospital of Alexandroupolis, Democritus University of Thrace, Alexandroupolis, Greece; ^8^ Department of Hematology, University Hospital of Larissa, Larissa, Greece; ^9^ Forth Department of Medicine, Aristotle University of Thessaloniki, “Hippokration” Hospital, Thessaloniki, Greece; ^10^ Department of Hematology, University of Crete School of Medicine, University Hospital of Heraklion, Heraklion, Greece; ^11^ Department of Hematology, “G. Gennimatas” General Hospital, Athens, Greece; ^12^ Hematology Unit, First Department of Internal Medicine, National and Kapodistrian University of Athens, Laikon General Hospital, Athens, Greece; ^13^ Hematology Laboratory, University General Hospital “Attikon”, Athens, Greece; ^14^ 2nd Department of Internal Medicine, Medical School, University of Athens, Hippokration General Hospital, Athens, Greece

**Keywords:** paroxysmal nocturnal hemoglobinuria (PNH), thrombosis, hemolytic anemia, orphan disease, complement inhibitors

## Abstract

Thrombosis is the most common and a life-threatening complication in patients with Paroxysmal Nocturnal Hemoglobinuria. One-third of patients with PNH experience at least one thromboembolic event during the course of the disease, with thrombosis being the most common cause of death in these patients. The mechanism of thrombosis in PNH is complex and continues to be of great research interest. Since the introduction of C5 complement inhibitors in the treatment of PNH, the incidence of thromboembolic events has decreased substantially. We retrospectively analyzed data concerning the thrombotic episodes of 41 patients with PNH from 14 different national hematology centers in Greece. Sixteen patients (39%) experienced at least one episode of thrombosis, including, seven (43.8%) at diagnosis, seven (43.8%) during the course of the disease and two (12.5%) patients prior to PNH diagnosis. Nearly half of these individuals (n=7, 43.8%) had multiple episodes of thrombosis during the course of their disease. The most common sites of thrombosis were intra-abdominal veins. Three out of 26 patients developed thrombosis while on eculizumab. In none of the 16 patients, the thrombotic event was fatal. Our findings, despite the small number of patients, confirmed that thrombosis continues to be a significant complication of PNH affecting more than one third of the patients.

## Introduction

Paroxysmal nocturnal hemoglobinuria (PNH) is a rare, potentially life-threatening, acquired clonal non-neoplastic hematopoietic stem cell disorder. PNH is characterized by complement-mediated chronic hemolytic anemia, acquired thrombophilia and bone marrow deficiency (1, 2).

The primary genetic defect in PNH comprises somatic mutations in the X-linked *PIGA* gene (2). *PIGA* encodes a protein that is involved in the biosynthesis of the glycosyl-phosphatidyl-inositol (GPI), a glycolipid that acts as an anchor that mediates attachment of over 150 proteins to cell membrane (GPI-anchored proteins) (2). Mutations in *PIGA* result in a significant reduction or lack of synthesis of the GPI anchor, with consequent partial or complete deficiency of GPI-anchored cell surface proteins such as the complement-inhibiting proteins, CD55 “(DAF - decay accelerating factor)” and CD59 (MIRL – membrane inhibitor of reactive lysis) (2, 3). CD55 by destabilizing the complement C3 convertase complex inhibits complement activation beyond C3 while CD59 exerts a direct inhibitory effect on the membrane attack complex (MAC), the final product of complement activation (2). Therefore, due to CD55 and CD59-deficient red cells, complement activation results in intravascular hemolysis and anemia (2).

In addition, PNH has been characterized as *“the most vicious acquired thrombophilic state known in medicine*” underscoring the high risk of thrombosis and the associated morbidity and mortality in untreated patients (4). The mechanisms of thrombosis in PNH are multifactorial and seem to involve complex interactions between the coagulation and complement cascades (5). It has been postulated that complement-mediated intravascular hemolysis and PNH platelet activation are also key pro-thrombotic factors (5). Other factors favoring a prothrombotic state include lack of nitric oxide bioavailability because of intravascular hemolysis, pro-inflammatory mediators, a dysfunctional fibrinolytic system, and damage to the endothelium (5). Patients diagnosed with PNH are at an increased risk for developing thrombosis, and clinical thrombotic episodes can occur in up to 45% of patients across all vascular sites (5, 6). Most thrombotic events are venous (85%) while arterial thrombosis is seen in 15% of cases and often affects the cerebral and coronary arteries (5, 6). Thrombosis is the leading cause of death in PNH, accounting for 40-67% of PNH deaths with known causes (5).

Thrombotic events (TE) necessitate initiation of anticoagulation treatment. However, anticoagulants alone are insufficient to prevent new TEs and therefore disease-specific therapy with complement inhibitors is indicated (4, 5, 7). Eculizumab, the first-in-class anti-C5 humanized antibody, has shown to decrease hemolysis and the frequency of serious and life-threatening morbidities such as thrombosis in PNH patients (8, 9).

In this study, we provide real-world data on TEs occurred in a cohort of 41 patients with PNH who were identified and treated over the past two decades.

## Patients and methods

We evaluated 41 patients diagnosed with PNH at 14 different hematology centers in Greece between 2002 and 2022. Only patients with adequate data were included. Their medical records were reviewed, and demographic and clinical data were collected. Symptoms and medical history, lab results at the time of diagnosis, and the size and type of PNH clone were all considered. The sites of thrombosis, the time of onset in relation to the course of the disease, any potential contributory factors, and the type and duration of anticoagulant therapy (primary or secondary thromboprophylaxis) were all evaluated. Blood transfusions requirements and other information on therapeutic interventions, whether disease-specific or not, were also assessed. Statistical analysis of the data was performed using the IBM SPSS 26 statistical package program. The chi-square test was used to determinate the relationship between groups and categorical variables. For the comparisons of numeric variables, independent samples *t*-tests were conducted. Differences were considered statistically significant when *p*-value was <0.05.

## Results

### Patient characteristics

We analyzed the demographic and clinical characteristics of 41 patients diagnosed with PNH. Twenty-two (53.7%) were male and nineteen (46.3%) female, with a median age at diagnosis of 33 (range, 11 - 82 years).

Twenty-three patients (56.1%) were identified with the classical form of PNH, three (7.3%) were diagnosed with the subclinical form, and fifteen patients (36.6%) were diagnosed in conjunction with another underlying disorder. The classification was based on the definitions of International PNH Interest Group (10). Seven (46.7%) of the latter group were diagnosed with aplastic anemia (AA), four (26.7%) with myelodysplastic syndrome (MDS), two (13.3%) with primary myelofibrosis (PMF), one (6.7%) with polycythemia vera (PV) and one patient had beta-thalassemia (6.7%).

In the study population, the median size of the GPI-deficient clone was 63% for granulocytes (range, 2.07-96.6%), 73% for monocytes (range, 2.47-98%), and 21.9% (range, 0.2-97%) for red cells (type II and III).

The most prevalent signs and symptoms at diagnosis were weakness (65.9%), abdominal discomfort (29.3%), hemoglobinuria (39%), back pain (7.3%), headache (4.9%), dysphagia (4.9%), and erectile dysfunction (4.5%). Splenomegaly was found in 19.5% of patients, whereas 22% (9/41) and 9.8% (4/41) of patients exhibited hepatic and renal impairment, respectively. At the time of diagnosis, 38 patients (92.7%) presented with anemia, while thrombocytopenia was noted in 26 patients (63.4%). Elevated LDH levels (LDH ≥1.5x the upper limit of normal [ULN]) were found in most of the patients (78% [32/41]) with a median value of 1,322 U/L (range, 399-4,024 U/L, normal value < 240 U/L). **Table 1** displays baseline demographic, clinical and laboratory data.

**Table 1:** Demographic, clinical and laboratory findings in PNH patients at diagnosis.

Parameter	All patients	Patients with TE	Patients without TE
Number and (%)	41 (100)	16 (39)	25 (61)
Median age, years (range)	33 (11–82)	43 (19–82)	32 (11-68)
Sex (M/F), n and (%)	22 (53.7)/19 (46.3)	7 (43.7)/9 (56.3)	15 (60)/10 (40)
Laboratory Findings, median and (range)			
Hemoglobin, g/dl	8.5 (4-14.4)	9.4 (5-14.4)	8.2 (4-12.3)
MCV, fL	96.1 (77.4-136)	87.2 (79.2-136)	98 (77.4-132)
WBC, K/μL	4.2 (1.8-17.2)	4.2 (2.5-10.9)	4.2 (1.8-17.2)
Platelets, K/μL	116 (3-740)	113 (18-740)	116 (3-342)
LDH, U/L	890 (156-4,024)	858 (242-4,024)	1,020 (156-3,716)
Flow Cytometry, median and (range)			
Granulocyte PNH clone, %	63 (2.07-96.6)	63 (2.6-96.6)	61 (2.07-95.1)
Monocyte PNH clone, %	73 (2.47-98)	60 (4.6-91.2)	76.8 (2.47-98)
Red cell type II+III PNH clone, %	21.9 (0.2-97)	33 (0.6-97)	20.4 (0.2-89.5)
Signs and Symptoms, n and (%)			
Anemia	38 (92.7)	15 (93.8)	23 (92)
Thrombocytopenia	26 (63.4)	10 (62.5)	16 (64)
Weakness	27 (65.9)	11 (68.8)	16 (64)
Hemoglobinuria	16 (39)	7 (43.8)	9 (36)
Abdominal discomfort	12 (29.3)	10 (62.5)	2 (8)
Splenomegaly	8 (19.5)	6 (37.5)	2 (8)
Hepatic dysfunction	9 (22)	8 (50)	1 (4)
Backpain/headache	3 (7.3)/2 (4.9)	2 (12.5)/2 (12.5)	1 (4)/0
Renal dysfunction	4 (9.8)	2 (12.5)	2 (8)
Dysphagia	2 (4.9)	1 (6.3)	1 (4)
Erectile dysfunction	1/22 men (4.5)	1/7 men (14.2)	0
PNH type, n and (%)			
Classical	23 (56.1)	11* (68.8)	13 (52)
Underlying Disease	15 (36.6)	5 (31.3)	10 (40)
*AA*	7 (46.7)	1 (6.3)	6 (24)
*MDS*	4 (26.7)	1 (6.3)	3 (12)
*PMF*	2 (13.3)	1 (6.3)	1 (4)
*PV*	1 (6.7)	1 (6.3)	0
*β-Thal*	1 (6.7)	1 (6.3)	0
Subclinical	3 (7.3)	0*	2 (8)
Survival status, n and (%)			
Alive	33 (80.5)	13 (81.2)	20 (80)
Dead	8 (19.5)	3 (18.8)	5 (20)

TE, Thrombotic Event; MCV, Mean corpuscular volume; WBC, White blood count; LDH, Lactate dehydrogenase; AA, Aplastic Anemia; MDS, Myelodysplastic Syndrome; PMF, Primary Myelofibrosis, PV, Polycythemia Vera; β-Thal, β-Thalassemia.

*One patient was diagnosed with subclinical PNH that progressed to the classical form over the years.

Sixty-one percent of patients had received red blood cell transfusions, whereas just six patients (14.6%) received platelet transfusions.

Regarding specific anti-complement treatment, 26 (63.4%) patients received eculizumab (treatment for 8 of them began with a TE), and 6 patients received ravulizumab (4 of them as second-line treatment and 2 as first-line therapy). The remaining 13 patients of our cohort did not receive any anti-complement therapy.

### Thromboembolic events

In total, 16 of the 41 patients (39%) experienced at least one TE, with venous thrombosis being the most common (68.8%). Five of the 16 patients (31.3%) had arterial thrombosis, and two of them (12.5%) also developed venous thrombosis. In 2 patients (4.9%), the TE preceded the PNH diagnosis by several years. Seven patients had thrombosis at the time of diagnosis, while the remaining seven experienced thrombosis later in the course of their disease. For these patients, the median time from PNH diagnosis to thrombosis was 65 months (range, 25-143 months).

### Characteristics of patients with thrombotic events

The majority of patients who experienced a TE (11/16) had classical PNH, while the remaining 5 (31.3%) were diagnosed with PNH in association with an underlying bone marrow disorder.

The median size of the PNH granulocyte, monocyte, and red blood cell (type II and III) clones in patients with TE was 63% (range, 2.6-96.6%), 60% (range, 4.6-91.2%), and 33% (range, 0.6-97%), respectively. Ten patients (62.5%) with TE had a granulocyte clone size more than 50% (**Table 1**).

Patients with thromboembolism had a significantly higher incidence of abdominal discomfort, splenomegaly and hepatic dysfunction compared to those without thrombosis (*p*<0.001, *p*=0.03 and *p*=0.001 for abdominal discomfort, splenomegaly, and hepatic dysfunction, respectively).

### Sites of thrombosis

Seven patients (43.8%) had hepatic vein thrombosis resulting in Budd-Chiari syndrome (BCS), seven patients (43.8%) were diagnosed with portal vein thrombosis and four patients (25%) experienced mesenteric vessel thrombosis. Each of the following sites was reported with thrombosis once: superior sagittal sinus, splenic vein, pulmonary artery, and leg deep vein.

**Figure 1:**
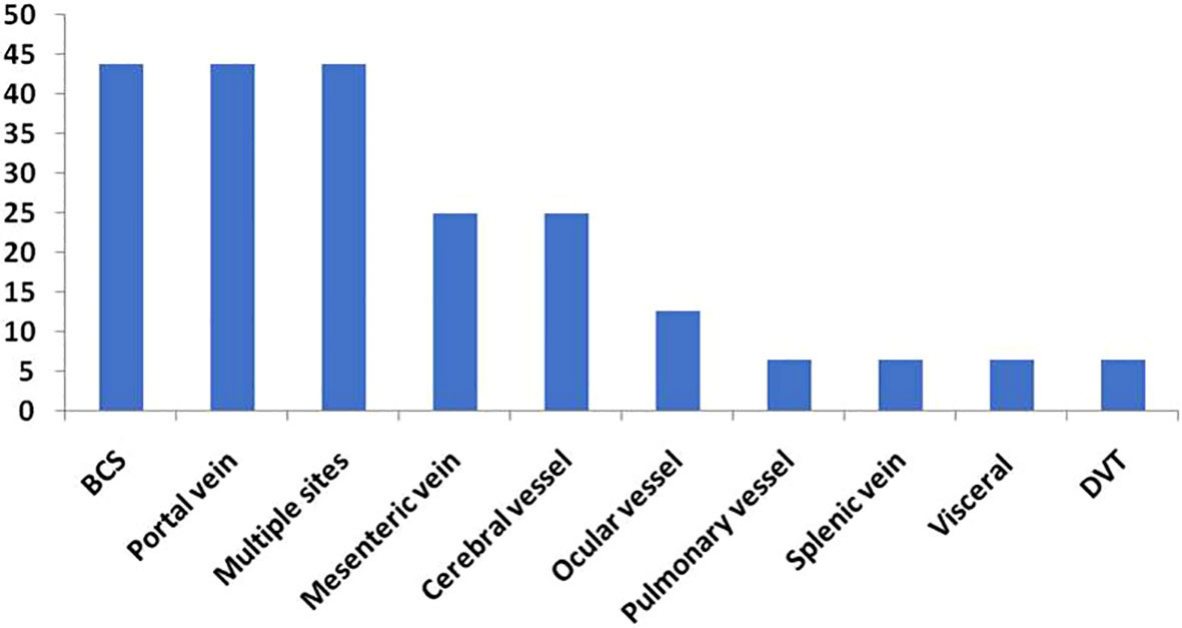
Sites of Thrombosis (%).

The distribution of arterial thrombosis was as follows: three patients had a stroke, one patient presented with thrombosis of the central artery of the left eye, and one patient had thrombosis at small ocular vessel, probably arterial (**Figure 1**).

### Patients with multiple thrombotic events

Seven out of sixteen patients (43.8%) for whom a detailed clinical picture is provided, experienced several TEs throughout the course of their disease.

*Patient #1* developed superior mesenteric and splenic vein thrombosis almost 12 years after her diagnosis, but she refused any other treatment, thus she received only anticoagulation therapy. *Patient #2* developed superior sagittal sinus and portal vein thrombosis 4.5 years after being diagnosed with PNH. One year after this episode, mesenteric vein thrombosis and BCS were identified and eculizumab therapy was initiated. *Patient #3* developed BCS and multiple visceral thrombosis of the liver, spleen, and left kidney, which led to the diagnosis of PNH shortly after being diagnosed with polycythemia vera. Two years after initiating treatment with eculizumab, the patient developed numerous cerebral vascular thromboses. *Patient #4* was initially diagnosed with subclinical PNH, which evolved to the classical form over time. Almost, 5.5 years later developed portal vein thrombosis and BCS. Eculizumab was administered shortly after the TE. *Patient #5* with a known myelodysplastic syndrome developed a mesenteric vein thrombosis and a cerebral stroke, and further investigation revealed the presence of a PNH clone. *Patient #6* developed mesenteric and portal vein thrombosis in addition to BCS, six years after being diagnosed with PNH. As complement inhibitors were unavailable at the time, she was treated solely with anticoagulants. *Patient #7* experienced portal vein and deep vein thrombosis (DVT) many years prior to the diagnosis of PNH; she was treated with low molecular weight heparin (LMWH) and started eculizumab as soon as PNH was detected.

### Management and treatment of TEs

None of the patients were undergoing prophylactic anticoagulant therapy at the time of the thrombosis. Anticoagulant treatment was applied to all patients who developed a TE. Most of them (13/16) were first treated with LMWH, whereas three received direct oral anticoagulants (DOACS), two apixaban and one rivaroxaban. Thirteen patients with TE (13/16) were treated with eculizumab; eight patients started upon diagnosis of TE and five patients after being diagnosed with PNH. Only three of the sixteen patients with thrombosis did not receive anti-complement therapy, either because drugs were unavailable when they were diagnosed or for other reasons (**Table 2**).

**Table 2:** Treatment of patients with TEs.

*n* of PNH pts with TE	16 7, upon diagnosis 7, later during the disease 2, long before diagnosis
*n* of PNH pts with TE treated with anticoagulants	16 13 LMWH/acenocumarol 3 DOACS
*n* of PNH pts with TE treated with anti-C5 inhibitor	13 8 with thrombosis initiating event of PNH 5 upon PNH diagnosis
*n* of PNH pts with TE not treated with anti-C5 inhibitor	3

TE, Thromboembolic event; pts, patients; LMWH, Low molecular weight heparin; DOACS, Direct oral anticoagulants.

### TEs in patients under treatment with eculizumab

Three out of 26 patients under treatment with eculizumab developed TEs. One patient with underlying PMF, 20 months after eculizumab initiation, experienced significant vision loss due to thrombosis in small ocular arterial vessel and he was switched to ravulizumab. Multiple cerebral vascular thromboses developed in the patient with concomitant PV while being two years on treatment with eculizumab. Lastly, the patient with homozygous thalassemia had a new thrombotic event almost two months after eculizumab treatment initiation for multiple previous thrombotic events (**Table 3**).

**Table 3:** TEs in relation to eculizumab treatment.

Without eculizumab treatment	n
Patients	13
Patients with TEs during the course of the disease	3
On eculizumab treatment	n
Patients	26
Patients with TEs during the course of the disease	3

TE, Thromboembolic event.

### TEs in patients who did not receive eculizumab

Thirteen (13/41) patients of our cohort did not receive eculizumab and three of them (3/13) developed TEs. One patient diagnosed with PNH/AA and developed pulmonary embolism and the other two diagnosed with classical type PNH and developed multiple thrombotic events in intra-abdominal sites (**Table 3**).

### Patient outcome

With a median follow-up of 68 months (range, 2-245 months), eight patients died and the median survival has not been reached. None of the TEs was fatal. Three patients experiencing thrombosis died from unrelated causes. One patient died from sepsis; another from severe COVID-19 several years after the TE; and the reason of death for one patient is unknown. The reasons of death for the patients without a history of TE were sepsis in two cases, secondary acute leukemia in one case, sudden death in one case, and severe COVID-19 in one situation.

## Discussion

All patients with PNH are at risk for thrombosis, and up to 45% of patients can experience clinical thrombotic episodes at both typical and atypical sites (5, 6). In the present study, 39% (16/41) of PNH patients experienced thrombosis during the course of their disease, a frequency comparable to that reported in the medical literature (5, 7, 9, 11).

In 17.1% (7/41) of the patients, the thrombotic episode was the primary cause of the PNH investigation. It is recognized that thrombosis in patients with PNH does not “respect” any blood vessel and can occur at any site (5). Intra-abdominal and cerebral veins are frequently affected by thrombosis for unknown reasons (5). In accordance with earlier findings, 29.3% (12/41) of our patients had intra-abdominal thrombosis.

In addition, it is anticipated that one-fifth of PNH patients develop thrombosis at numerous sites (5). Seven people (17.1%) in our study presented with thrombosis at several sites.

Unusual venous thrombotic events, intra-abdominal or Budd-Chiari syndrome, and cerebral vein thrombosis account for two-thirds of the thrombotic episodes (5, 6). Seven patients in our cohort were diagnosed with Budd-Chiari syndrome, which corresponds to 17.1% of all patients examined and 43.8% of patients who experienced TEs. Patients with Budd-Chiari syndrome primarily complained of abdominal discomfort, which was considerably worse in patients with a TE than in patients without a TE (*p*<0.001). Hepatic dysfunction and splenomegaly were observed to be substantially more prevalent in patients with a TE (*p*=0.001 and *p*=0.03 respectively). Ocular involvement is a rare manifestation of thrombosis in patients with PNH (5). Two patients, or 12.5% of all patients who experienced a TE, developed ocular thrombosis in our study. Both patients suffered serious vision loss as a result of these thrombi, which had catastrophic effects on their quality of life.

Several other hematological disorders, including aplastic anemia and, to a lesser extent, myelodysplastic syndrome, have been linked to the presence of a PNH clone (2, 12–15). In contrast, the combination of PNH and myeloproliferative diseases (MPN) is rare and is mostly associated with primary myelofibrosis (13–17). The existence of a PNH clone in PV is extremely uncommon and especially challenging to detect when an intra-abdominal thrombosis occurs. In fact, less than 40 cases have been described in the literature to date where PNH may be associated with a MPN (12, 17–19). In our dataset, four (26.7%) individuals had concomitant MDS, two (13.3%) had primary myelofibrosis and one (6.7%) had PV. The PV patient is a female who was diagnosed with the coexistence of PNH clone and a JAK2V617F-positive PV, with atypical thromboses and not overt hemolysis (18).

It is generally accepted that the size of the PNH clone is one of the most significant risk factors for thrombosis (5, 8, 9, 20–22). In this study, the median size of the granulocyte and monocyte PNH clone in patients with a thrombotic episode was 63% (range, 2.6-96.6%) and 60% (range, 4.6-91.2%) respectively. Patients with PNH and >50% granulocyte clones have a ten-year risk of venous thrombosis of 44%, according to previous findings (9, 23). In patients with a smaller clone size, this proportion was only 5.8%, which is still higher than what is observed in healthy subjects (9, 23). In our study, among 16 patients with a thrombotic event, 10 (62.5%) patients had granulocyte clone sizes greater than 50%.

Elevated LDH at diagnosis is associated with an increased risk of thrombosis (24, 25). In our study group, the median laboratory values of baseline LDH was increased in both patients with and without a TE (858 U/L, range 242-4,024 U/L and 1,020, range 156-3,716 U/L respectively) with no significant difference between the two groups (*p*=0.83).

It is reasonable to initiate prophylactic anticoagulant therapy at the time of PNH diagnosis; but, long-term anticoagulants are an additional burden for PNH patients and pose a risk of bleeding for half of individuals who will never develop thrombosis (4, 9). No patient in our study received preventive anticoagulant therapy.

Anticoagulants such as heparin or LMWH are still the preferred treatment for acute thrombotic events in PNH patients (26). DOACs are probably equivalent, but their use in PNH has not been exhaustively investigated (26). However, it is widely recognized that anticoagulants alone could not control thrombosis for an extended period of time (6, 26). This requires treating TEs with a complement blocker, which targets the thrombus’ underlying etiology. To prevent the activation of the complement system, eculizumab, ravulizumab or another approved complement inhibitor should be administered with the anticoagulants as soon as possible, if they are available (6, 26). Eculizumab, the first-in-class anti-C5 humanized antibody, improves patient outcome by reducing hemolysis and the incidence of life-threatening morbidities, such as thrombosis, in PNH patients (8, 9). Long-acting C5 inhibitor Ravulizumab is non inferior to eculizumab in terms of efficacy, safety, and tolerance (27–29). Pegcetacoplan is the first C3 inhibitor approved for adult PNH treatment. Both extravascular and intravascular hemolysis are prevented. Anti-C3 compounds, anti-factor D agents, and anti-factor B agents are the strategic targets of many other newly developed therapeutics (30, 31).

The majority of patients examined in our study received LWMH as anticoagulant therapy after the thrombotic episode, with six patients receiving acenocoumarol shortly after LWMH treatment, and three started on DOACs. According to international guidelines, eight patients were started on eculizumab immediately after the TE. Although a considerable reduction in TEs has been demonstrated, the risk exists for patients receiving eculizumab (6, 22, 32). In our cohort, 3 of 13 patients with PNH who did not receive eculizumab treatment developed a TE during the course of the disease, whereas 3 of 26 patients treated with eculizumab were reported to have TEs. Although we have no information regarding the possible cause of thrombosis in patients treated with eculizumab, administration delays may be an issue, but additional information is required. Notably, each of those three patients had a concomitant underlying disease, one PMF, one PV and one homozygous thalassemia.

Due to a lack of evidence on the safe duration of anticoagulant therapy following a thrombotic event in complement inhibitor-treated patients, the decision to discontinue should be made on a case-by-case basis and addressed with the patient (26, 33, 34). No data exist regarding the discontinuation of anticoagulants in our patient group.

In addition to anti-complement mAbs, hematopoietic stem cell transplantation (HSCT) has a curative role but is related to serious or fatal complications in PNH patients; hence, it can be considered for only certain cases, such as patients with severe cytopenias or patients with severe hemolysis or thrombosis refractory to complement inhibition (35). Even in these cases, eculizumab can be utilized to minimize morbidity prior to and during HSCT, as demonstrated by several case reports (36).

PNH is an acquired thrombophilic disorder, and the major cause of death is thrombosis, particularly in the pre-eculizumab era (5–7, 37). Further research indicates that the prevalence of PNH thrombosis has been greatly underestimated. An MRI finding of subclinical pulmonary embolism or early cardiac scarring in 60% and 20% of a small cohort of young PNH patients, respectively, supports this (5, 38). In our study, however, none of the 16 patients who had PNH-induced thrombosis died as a result of the TE. Of course, there were two deaths in our cohort for unspecified reasons, which may imply a possible pathogenetic correlation.

Our study has the limitations of a retrospective study with small number of patients. However, due to the rarity of the disease, prospective studies are difficult to conduct. Moreover, as this is a multicenter study, the cases reported represent a significant number of cases diagnosed and treated in Greece.

In conclusion, regardless of the small sample size, our study demonstrated that thrombosis is a serious consequence of PNH that affects more than one-third of patients. The majority of thrombotic episodes occurred at unusual sites. In addition, the study reveals that the incidence of thrombosis and its link with the size of the PNH clone are similar with prior findings. Ongoing treatment with complement inhibitors has considerably reduced the risk of thrombosis, although it has not eliminate it totally.

## Data availability statement

The original contributions presented in the study are included in the article/supplementary material. Further inquiries can be directed to the corresponding author.

## Author contributions

SC collected, analyzed the data and wrote the manuscript. MA, JVA, NEL, TC, PZ, AB, ZB, EK, CF, PC, DS, VD, ASi, EM, MPs, HAP, TM, NAV, SK, FK, ASk, GV, IK, VP, CL, IB, SD, and MPag provided data. EH, MPap designed the study, provided, analyzed the data and wrote the manuscript. All authors contributed to the article and approved the submitted version.
